# Blood expression levels of chemokine receptor CCR3 and chemokine CCL11 in age-related macular degeneration: a case–control study

**DOI:** 10.1186/1471-2415-14-22

**Published:** 2014-02-27

**Authors:** Mads Krüger Falk, Amardeep Singh, Carsten Faber, Mogens Holst Nissen, Thomas Hviid, Torben Lykke Sørensen

**Affiliations:** 1 Clinical Eye Research Unit, Department of Ophthalmology, Copenhagen University Hospital Roskilde, Denmark and Faculty of Health Sciences, University of Copenhagen, Køgevej 7-13, DK-4000 Roskilde, Denmark; 2 Department of Microbiology, Immunology & International Health, Faculty of Health Sciences, University of Copenhagen, Copenhagen, Denmark; 3 Department of Clinical Biochemistry, Center for Immune Regulation and Reproductive Immunology, Copenhagen University Hospital Roskilde, Denmark and Faculty of Health Sciences, University of Copenhagen, Copenhagen, Denmark

**Keywords:** Age-related macular degeneration, chemokine, CCR3, CCL11

## Abstract

**Background:**

Dysregulation of the CCR3/CCL11 pathway has been implicated in the pathogenesis of choroidal neovascularisation, a common feature of late age-related macular degeneration (AMD). The aim of this study was to investigate the expression of CCR3 and its ligand CCL11 in peripheral blood in patients with neovascular AMD.

**Methods:**

Patients with neovascular AMD and healthy controls were included. Blood samples were obtained and prepared for flow cytometry to investigate the expression of CCR3. Levels of CCL11 were measured in plasma using Cytometric Bead Array. Differences between the groups were tested using Kruskal-Wallis test and Mann–Whitney U test.

**Results:**

Patients (n = 83) with neovascular AMD and healthy control persons (n = 114) were included in the study. No significant difference in the expression of CCR3 was found on CD9+ granulocytes when comparing patients suffering from neovascular AMD with any of the control groups. We did not find any alteration in CCL11 levels in patients among the age matched groups. There was no correlation between expression of CCR3/CCL11 and clinical response to treatment with anti-vascular endothelial growth factor (VEGF).

**Conclusion:**

Our results do not suggest a systemic alteration of the CCR3/CCL11 receptor/ligand complex in patients with neovascular AMD.

## Background

Age-related macular degeneration (AMD) is the leading cause of blindness among elderly people in the developed part of the world [1]. The late stage of AMD presents in two distinct forms: Geographic atrophy (GA) and neovascular AMD [2]. The introduction of anti-vascular endothelial growth factor (VEGF) treatment has drastically improved the course of neovascular AMD, but the need for developing improved therapies is still warranted [3].

The growth of a choroidal neovascularisation (CNV) is probably mainly driven by VEGF, but clinical studies and laboratory data have suggested that other potential signal molecules, including chemokines, could be involved in CNV growth [4-7]. In particular there has been a growing body of evidence suggesting an involvement of the chemokine receptor CCR3 in CNV development [4,5,8]. Chemokines are released from sites of inflammation to recruit cells that express chemokine receptors [9]. The chemotactic function was previously believed to be the main function of chemokines, but in the recent years several other functions of the different chemokines and chemokine receptors have been proposed. CCR3 is a G protein coupled chemokine receptor. It is expressed mainly on CD9+ eosinophil granulocytes and basophil granulocytes, but also on Th2-cells. CCR3 is known for its role in allergic diseases like allergic asthma, allergic rhinitis, atopic dermatitis and may function by recruiting and activating leukocytes at sites of inflammation [10]. CCR3 is also found on human endothelial cells [8].

In AMD, Takeda *et al*. [4] found that CCR3 was expressed in choroidal endothelial cells, only in patients with CNV, and in the same report laser-induced CNV formation in mice was blocked with anti-CCR3 treatment. In addition CCR3 expressing cells have been demonstrated in post-mortem eyes from patients with neovascular AMD. In an experimental study of human choroidal endothelial cells Wang *et al*. found that CCR3 expression was increased in aging eyes [5]. Furthermore this study reported that CCR3, when activated by the ligand CCL11, increased migration of choroidal endothelial cells, whereas it was inhibited by a CCR3 inhibitor [5]. In a recent study Sharma *et al*. showed a significant association between AMD and a single nucleotide polymorphism (SNP) in the CCR3 gene [11].

CCR3 binds a variety of C-C chemokines one of which is CCL11 (formerly known as eotaxin). CCL11 is best known for its ability to mediate allergic responses and for its ability to attract eosinophil granulocytes [12], however several studies in mice have elucidated other potential roles for CLL11, since an age dependant increase in circulating CCL11 has been shown [13,14]. Importantly Salcedo *et al*. reported some years back that CCL11 is involved in angiogenesis [8]. In one study on circulating CCL24/eotaxin 2, another ligand for CCR3, Sharma *et al*. found an increased level of circulating eotaxin 2 in peripheral blood in patients with AMD compared to controls [15].

We have previously found altered expression on peripheral blood leukocytes in patients with AMD, among others an increased expression of CD200 on peripheral blood monocytes in patients with neovascular AMD [16].

Since CCR3 has been so strongly implicated in CNV formation, it could be a target for treatment. However the correlation between CNV and CCR3 has mainly been shown in murine models of CNV. Therefore, we studied the expression of CCR3 on CD9+ granulocytes and CD4+ T-cells in peripheral blood of patients with different clinical stages of AMD, and measured the plasma levels of the CCR3 ligand CCL11. This is the first study to investigate the association between the peripheral CCR3/CCL11 receptor/ligand complex and AMD.

## Methods

### Participants

Patients with neovascular AMD attending our department were asked to participate in this case–control study. Patients attending our department for other reasons were included as control subjects and divided in two groups according to age: one group containing persons 70 years or older and one group containing persons less than 70 years old. Patients were excluded from the study if they had been diagnosed with autoimmune or malignant disease, and patients were also excluded if they were receiving immune modulating therapy. To avoid interference with other acute-phase responses due to infections, or to avoid interference with potentially undiagnosed cancer, we excluded post hoc any participant with a C-reactive protein (CRP) above 10 mg/L.

All participants underwent a structured interview aiming at investigating previously and current medical conditions and medication status. Smoking habits and alcohol consumption were recorded. Patients were defined as current smokers, former smokers (more than 100 cigarettes during their lifetime) or never smokers. Alcohol consumption was recorded and graded according to the Danish National Board of Health’s recommendations (maximum 7 units and 14 units per week for women and men, respectively). Height and weight was measured to calculate the body mass index (BMI).

Verbal and written informed consent was obtained from all participants prior to inclusion in the study. The study has been approved by the Regional Committee of Ethics in Research of the Region of Zealand (SJ-142) and was performed in adherence to the Declaration of Helsinki.

### Ophthalmic examination

All patients included were examined with slit-lamp biomicroscopy and fundoscopy, Spectral-Domain-Optical Coherence Tomography (SD-OCT) (Spectralis HRA-OCT, Heidelberg Engineering, Heidelberg, Germany), and Fundus Autofluorescense Imaging. For exact diagnosis fluorescein- and indocyanine green (ICG) angiography (FA/ICG) was performed on all patients suspicious of neovascular AMD. Visual acuity (VA) was measured using the Early Treatment Diabetic Retinopathy Study (ETDRS) chart.

### Leukocyte preparation and flow cytometry

Venous blood samples were collected for flow cytometry and CCL11 quantification from all patients prior to angiography to avoid possible interference [17]. The blood samples were prepared within 4 hours of phlebotomy for flow cytometry by the following steps. Red blood cell lysis was performed by adding 10% red blood cell lysis buffer (Nordic Biosite 3AB, Täby, Sweden) to the whole blood for 10 minutes at room temperature. After red blood cell lysis the sample was washed three times in isotonic buffer (IsoFlowTM Sheath Fluid Beckman Coulter, Brea, CA, USA) and centrifuged for five minutes at 500G. After washing, the cells were incubated at room temperature in the dark for 25 minutes with the following monoclonal anti-human antibodies: CD9 (IgG1, Clone: ALB6, Beckman Coulter, Brea, CA, USA), CD4 (IgG1, clone: 13B8.2 Beckman Coulter, Brea, CA, USA) and CCR3 (IgG2a, clone: 61828, R&D Systems, Minneapolis, MN, USA) Corresponding negative isotype controls were used and set at 1%. Flow cytometry was performed on a Beckman Coulter FC 500 (Beckman Coulter, Brea, CA, USA) flow cytometer.

**Figure 1:**
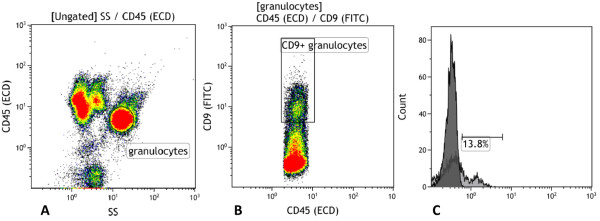
**The CCR3 expression was measured using flow cytometry.** The figure shows the gating strategy and quantification of CD9 positive granulocytes and their expression of CCR3. **A**, Granulocytes were identified based on CD45 expression and side scatter. **B**, the granulocytes were then gated based on the CD9 expression. **C**, the fraction of CCR3+ cells within the CD9+ granulocyte gate was calculated. Histogram showing the percentage of CD9 positive granulocytes positive for CCR3 (Light grey) and the negative isotype control set at 1% (dark grey).

The gating strategy for CD9 positive CCR3 positive cells can be seen in Figure 1.

### CCL11 quantification

Blood samples were centrifuged for 15 minutes at 1000 G, and plasma was isolated and stored at -80 degrees Celsius until analysis were performed. CCL11 was measured using a Cytometric Bead Array (BD biosciences, Franklin Lakes, NJ, USA) following the manufactures recommendations.

### Clinical response

To assess the clinical relevance of peripheral expression of CCR3 and CCL11 we looked at treatment response after the initial three injections with Ranibizumab (Lucentis®, Genetech, San Francisco, USA), since it has been published that VA at this point of treatment is a strong predictor of long term treatment response [18]. Patients in active treatment with Ranibizumab were divided in groups according to their change in VA from the first visit to the follow up visit. The patients were first divided in three groups according to a change in VA of 10 ETDRS letters or more, and secondly according to a change in VA of 15 ETDRS letters or more.

### Statistical analysis

The statistical software SPSS® version 19 for Windows (IBM, Chicago, IL USA) was used for statistical analysis. Kruskal-Wallis test and Mann–Whitney U test were used for not normally distributed continuous variables (CCR3, CCL11, age, BMI) and data are given as medians and interquartile ranges (IQR). Categorical variables were analyzed using Pearson Chi-square test (Gender, alcohol-intake, smoking habits). Correlation between variables was tested using Spearman correlation test. A p-level of less than 0.05 was considered significant.

## Results

The chemokine receptor CCR3 was measured in 159 patients and the chemokine CCL11 was measured in 197 patients. The demographic and clinical data can be seen in Tables 1 and 2. There was no significant difference in male/female ratio, smoking habits, alcohol consumption, or BMI between the groups. There was a significant difference in age between the groups. This difference disappeared when the group of young control persons was excluded.

**Table 1:** Demographic data and clinical characteristics of patients included in the study

	**nAMD (n = 77)**	**Controls > 70 years (n = 18)**	**Controls < 70 years (n = 64)**	**P-value**	**Test**
Median Age in Years (min-max)	75.2 (60–92)	77.0 (70–89)	55.6 (32–69)	0.0001	Kruskal-Wallis test
Male %	36	39	42	0.702	Pearson Chi-square test
Female %	64	61	58		
Smoking habits:					
Current smokers, (% yes)	19	12	19	0.956	Pearson Chi-square test
Former smokers (% yes)	48	44	44		
Never smokers (% yes)	33	44	37		
Alcohol intake					
Above recommended (% yes)	16	17	13	0.159	Pearson Chi-square test
BMI (median, IQR), kg/m^2^	24.4 (22.7;28.6)	26.1 (24.1;29.3)	24.9 (22.0;29.7)	0.506	Kruskal-Wallis test
Percentage of CD9 positive granulocytes expressing CCR3. (Median, IQR)	5.25 (2.8;9.1)	3.8 (2.0;6.4)	5.8 (2.6-5.8)	0.234	Kruskal-Wallis test

The table shows Demographic data and clinical characteristics of patients in whom CCR3 expression was measured. Difference among groups is calculated using Kruskal-Wallis test and Pearson Chi square test. P-values are given.

*AMD* Age-related Macular Degeneration, *nAMD* neovascular AMD, *IQR* Inter Quartile Range, *BMI* Body Mass Index.

**Table 2:** Demographic data and clinical characteristics of patients included in the study

	**nAMD (n = 83)**	**Controls > 70 years (n = 24)**	**Controls < 70 years (n = 90)**	**P-value**	**Test**
Median Age in Years (min-max)	76.0 (63–92)	76.0 (70–85)	53.0 (30–69)	0.001	Kruskal-Wallis test
Male %	45	38	37	0.260	Pearson Chi-square test
Female %	55	62	63		
Smoking habits:					
Current smokers, (% yes)	24	13	14	0.650	Pearson Chi-square test
Former smokers (% yes)	40	33	41		
Never smokers (% yes)	34	50	43		
Alcohol intake					
Above recommended (% yes)	17	14	13	0.646	Pearson Chi-square test
BMI (median, IQR), kg/m^2^	25.5 (23.0;28.3)	26.1 (22.3;27.9)	24.5 (21.9;28.5)	0.757	Kruskal-Wallis test
Plasma concentration of CCL11 (median, IQR) pg/ml	516 (342;721)	418 (275;840)	361 (240;503)	0.001	Kruskal-Wallis test

The table shows Demographic data and clinical characteristics of patients in whom plasma concentration of CCL11 was measured. Difference among groups is calculated using Kruskal-Wallis test and Pearson Chi square test. P-values are given.

*AMD* Age-related Macular Degeneration, *nAMD* neovascular AMD, *IQR* Inter Quartile Range, *BMI* Body Mass Index.

**Figure 2:**
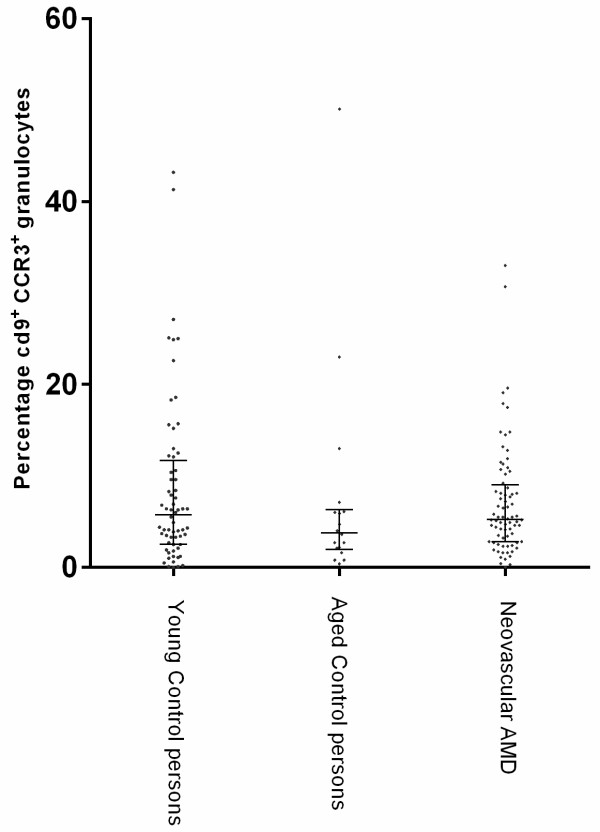
**Percentage of CD9+ cells expressing CCR3 in young controls (< 70 years), aged controls (>70 years) and patients with neovascular AMD.** Each dot represents one patient. The lines denote medians and interquartile ranges (IQR). No significant difference was found between the groups.

No significant change in percentage CD9 positive granulocytes expressing CCR3 was found when comparing the group with neovascular AMD with any of the control groups (Figure 2 and Table 1). No correlation was found when comparing the expression of CCR3 on CD9 positive cells with smoking habits, BMI or physical activity. No difference in expression of CCR3 on CD4 positive cells was found among the groups (data not shown).

**Figure 3:**
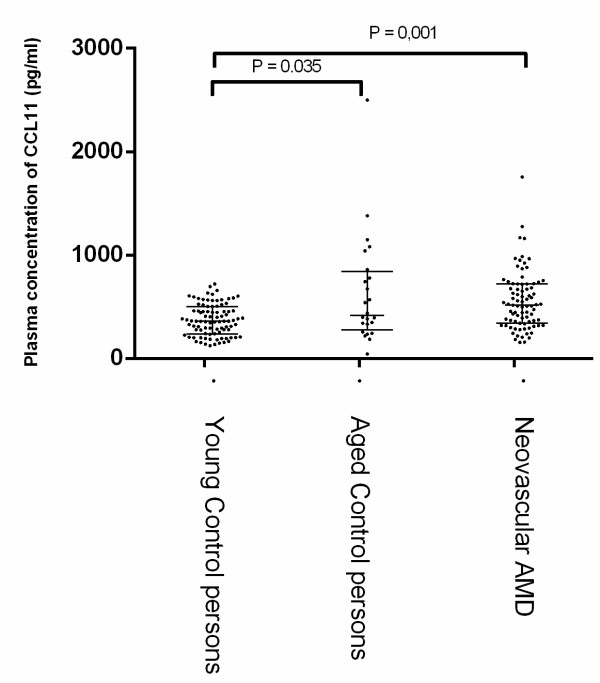
**Plasma concentrations of CCL11 in young controls (< 70 years), aged controls (>70 years) and patients with neovascular AMD.** Each dot represents one patient. The lines denote medians and interquartile ranges (IQR). Significant differences between groups are marked with p-values, Mann–Whitney U Test.

Table 2 and Figure 3 shows the plasma levels of CCL11. There was an expected age related difference in the plasma level of CCL11 between the group of young control persons and the group of old control persons (P = 0.035). Furthermore, we found a significant increase in the CCL11 level in patients with neovascular AMD (p = 0.001) when comparing this group with the group of young control persons. No difference was found when the group of patients with AMD was compared with the age matched control group.

To assess the clinical relevance of the peripheral plasma concentration of CCL11 level of CCR3 expression we checked the change in VA after the three initial injections with Ranibizumab. We divided the patients with AMD in groups according to a change in VA as described previously. Table 3 shows the plasma levels of CCL11 and the percentage of CD9 positive granulocytes expressing CCR3 among the different groups. We did not find any significant difference in plasma level of CCL11 or expression of CCR3 among the three groups when looking at a VA change of minimum 10 ETDRS letters . We also looked at clinical meaningful change in VA defined as a change in VA of at least 15 ETDRS and did not find any statistical significant difference among the groups.

**Table 3:** Plasma level of CCL11 or expression of CCR3 according to change in visual acuity

	**Gain of minimum 10 ETDRS letters (n = 10)**	**VA change of less than 10 ETDRS letters (n = 56)**	**Loss of minimum 10 ETDRS letters (n = 16)**	**P-value Kruskal-Wallis test**
CCL11 plasma level Median (IQR)	473.6 (408.9;580.7)	534.1 (323.0;710.6)	427.7 (243.7;762.5)	0.725
CCR3 positive granulocytes Median (IQR)	4.85 (3.18;6.08)	5.50 (4.13;9.67)	7.30 (3.18;10.7)	0.530
	**Gain of minimum 15 ETDRS letters (n = 7)**	**VA change of less than 15 ETDRS letters (n = 64)**	**Loss of minimum 15 ETDRS letters (n = 11)**	
CCL11 plasma level Median (IQR)	432,3 (390.5;635.8)	541.2 (351.4;699.5)	349.2 (240.7;667.1)	0.382
CCR3 positive granulocytes Median (IQR)	4.60 (2.65;6.90)	5.50 (4.13;9.67)	8.00 (3.65;10.60)	0.496

The table shows the plasma level of CCL11 and expression of CCR3 among patients with neovascular AMD in active treatment with anti-VEGF. The clinical relevance of CCL11 and CCR3 was tested by dividing the patients in groups according to change in visual acuity (VA). First we tested by dividing the patients in three groups according to a change in VA of minimum 10 ETDRS letters and secondly by dividing the patients in three groups according to a VA change of minimum 15 ETDRS letters. Difference among groups is calculated using Kruskal-Wallis test. P-values are given.

*IQR* Inter Quartile Range, *BMI* Body Mass Index, *ETDRS* Early Treatment Diabetic Retinopathy Study.

## Discussion

The pathogenesis of AMD remains largely unknown. Different pathogenic mechanisms have been suggested in development of AMD. Formation of CNV is the hallmark of late stage neovascular AMD. It is generally accepted that CNV formation is mainly driven by VEGF but other factors have proven to be involved in the formation of CNV [19].

The chemokine receptor CCR3 and its ligand CCL11 have shown to be involved in angiogenesis. One mechanism of involvement could be through Tryptase-chymase double-positive human mast cells expressing CCR3 [20].

In an experimental study on human endothelial cells Salceda *et al*. found that cells expressing CCR3 induced an angiogenic response when exposed to the ligand CCL11 [8]. Several studies have suggested the involvement of CCR3 and its ligands in the development of AMD and CNV [4,5,21]. Different murine models of CNV have formed a part of the investigation of the role of CCR3 in CNV formation. Most of these studies point in the same direction that CCR3 plays a role in the development of CNV [4,5,21]. Ahmed *et al*. used a murine model to study the role of Notch signalling in ocular angiogenesis. They found an increase in transcription level of the pro-angiogenic factor VEGFR2 and CCR3 in subjects with CNV compared to controls [22]. However Li *et al*. investigated the CCR3 in relation to CNV also in a murine model of CNV and found no evidence of the involvement of CCR3 in CNV formation; in fact they could not find any specific expression of CCR3 in or near the CNV [23]. One reason for this could be that the mouse model used in this study did not exert oxidative stress on the retinal pigment epithelium (RPE) as suggested by Wang *et al*. In an experimental study of human choroidal endothelial cells Wang *et al*. found that CCR3 was only expressed in the choroidal endothelial cells under certain conditions such as increased age and oxidative stress. Oxidative stress is a well known factor in the development of AMD and CNV and Wang *et al*. showed that when the retinal sections were exposed to oxidative stress the response was an increased expression of CCR3 in the choroidal endothelial cells. Furthermore when these cells were stimulated with CCL11 it induced a choroidal endothelial cell migration [5]. Overall there is increasing evidence for the involvement and importance of CCR3 in CNV formation when expressed locally on the endothelial cells in the choroid. Evidence points toward an increased level of CCR3 expression on choroidal endothelial cells in patients with neovascular AMD. We hypothesized that the peripheral expression of CCR3 would reflect the local CCR3 expression in the choroid. We studied the expression of CCR3 on peripherally CD9 positive eosinophiles and CD4 positive t-cells in patients with neovascular AMD and healthy control persons. We did not find any difference in expression of CCR3 between the AMD-group and the control groups. It is well known that chemokines and chemokine receptors have diverse function locally and systemically. This means that a locally increased expression is not necessarily reflected systemically. Since increasing evidence points towards CCR3 as an important factor in developing CNV our finding does not rule out the possible involvement of CCR3 and it ligand CCL11 in CNV formation. Our results show that the peripheral CCR3 expression level is not altered in patients with neovascular AMD compared to controls. However, the study is limited by its relatively small number of patients included and its institutional study design. Furthermore, we have not measured the CNV size prior to treatment. It is well known that treatment response is related to CNV size. Most of the studies that have investigated the role of CCR3 in CNV formation have been experimental studies or studies on murine models of CNV. Our study differs from the other studies being an observational study on patients from a clinical setting.

Wang *et al*. found evidence that there might be cross-talk between CCR3 and VEGF. The study showed that treating choroidal endothelial cells with CCL11 activated VEGFR2 - the receptor for VEGF [5]. This is in line with findings from several other studies where blockade of CCR3 led to suppression of CNV formation [4,21]. In a recent study Sharma *et al*. showed a significant association between AMD and increased plasma level of eotaxin 2/CCL24, however the control group was significantly younger than the AMD group. The difference in CCL24 described by Sharma *et al*. might be due to the control group being 12 years younger than the AMD group, since there is evidence that plasma level of CCL11 changes with age [11].

We observed an age dependent increase in plasma concentration of CCL11 which is in concordance with findings in other studies [24,25]. We found a significant increase in plasma CCL11 levels in patients with neovascular AMD compared to young control persons, but did not find any difference in CCL11 plasma concentration when comparing the group of patients with neovascular AMD with the age matched control group. This is in contrast to the findings by Mo *et al*. who proposed CCL11 as a possible biomarker for AMD. In a study including 60 AMD patients and 20 controls they found the plasma level of CCL11 increased in all stages of AMD except neovascular AMD [25].

## Conclusion

CCR3 and its ligand CCL11 have been shown to play a potential role locally in the eye in the development of CNV. This is the first study to investigate the association between the peripheral expression of CCR3 and concentration of CCL11 and AMD. We did not find alterations in CCR3/CCL11 expression in peripheral blood in patients with neovascular AMD suggesting that the peripheral expression of the CCR3/CCL11 receptor/ligand complex plays a limited role in CNV formation in humans. When looking at Ranibizumab treatment response in terms of change in VA we found no significant correlation with CCR3 and CCL11 expression. However it is well known that chemokines and chemokine receptors have diverse function systemically and locally and our findings do therefore not rule out a potential role of CCL11 and CCR3 expression in the choroid in the formation of CNV.

## Abbreviations

AMD: Age-related macular degeneration; nAMD: neovascular AMD; IQR: Inter quartile range; BMI: Body mass index; CNV: Choroidal neovascularisation; VEGF: Vascular endothelial growth factor; GA: Geographic atrophy; CRP: C-reactive protein; SD-OCT: Spectral-domain-optical coherence tomography; FA: Fluorescein angiography; ICG: Indocyanine green; ETDRS: Early treatment diabetic retinopathy study.

## Competing interests

The authors declare that they have no competing interests.

## Authors’ contributions

TLS, AS, TH and MKF participated in the design of the study. AS, CF and MKF participated in the laboratory work. AS and MKF were responsible for inclusion of participants. MKF performed the statistical analysis and made the draft manuscript. All authors read and approved the final manuscript.

## Pre-publication history

The pre-publication history for this paper can be accessed here:

http://www.biomedcentral.com/1471-2415/14/22/prepub
